# Toward a socio-spiritual approach? A mixed-methods systematic review on the social and spiritual needs of patients in the palliative phase of their illness

**DOI:** 10.1177/02692163211010384

**Published:** 2021-04-20

**Authors:** Tom Lormans, Everlien de Graaf, Joep van de Geer, Frederieke van der Baan, Carlo Leget, Saskia Teunissen

**Affiliations:** 1 Center of Expertise Palliative Care Utrecht, UMC Utrecht, Utrecht, the Netherlands; 2 Academic Hospice Demeter, De Bilt, the Netherlands; 3 University of Humanistic Studies, Utrecht, the Netherlands

**Keywords:** Palliative care, pastoral care, spirituality, social behavior, social support, needs assessment, systematic review

## Abstract

**Background:**

Patients express a variety of needs, some of which are labeled social and spiritual. Without an in-depth exploration of patients’ expressions of these needs, it is difficult to differentiate between them and allocate appropriate healthcare interventions.

**Aim:**

To gain insight into the social and spiritual needs of patients with a life-limiting illness and the distinction between these needs, as found in the research literature.

**Design:**

A mixed-methods systematic review and meta-aggregation were conducted following the Joanna Briggs Institute (JBI) approach to qualitative synthesis and the PALETTE framework and were reported according to the PRISMA statement. This review was registered in PROSPERO (CRD42019133571).

**Data sources:**

The search was conducted in PubMed, EMBASE, CINAHL, Scopus, and PsycInfo. Eligible studies reported social and spiritual needs from the patients’ perspective and were published between January 1st 2008 and October 2020. The quality of evidence was assessed using JBI Critical Appraisal Tools.

**Results:**

Thirty-four studies (19 qualitative, 1 mixed-methods, and 14 quantitative) were included. The five synthesized findings encompassing social and spiritual needs were: being autonomous, being connected, finding and having meaning, having a positive outlook, and dealing with dying and death.

**Conclusion:**

What literature labels as social and spiritual needs shows great similarities and overlap. Instead of distinguishing social from spiritual needs based on patients’ linguistic expressions, needs should always be explored in-depth. We propose a socio-spiritual approach that honors and preserves the multidimensionality of patients’ needs and enables interdisciplinary teamwork to allocate patient-tailored care.

**What we already know on the topic?**

- Patients express multidimensional needs in the palliative phase of their illness, some of which are labeled as social and spiritual needs;
- When needs are labeled as social or spiritual, it is often unclear on what grounds;
- Hence, healthcare professionals experience challenges concerning time, knowledge, and experience in identifying, understanding, and addressing social and spiritual needs.

**What this paper adds?**

- This mixed-methods review is the first study combining qualitative and quantitative studies to distinguish between social and spiritual needs of patients in the palliative phase of their illness;
- Identifying needs through a socio-spiritual approach offers the possibility to respect the social and spiritual dimensions as both distinct and interrelated.

**Implications for practice, theory, or policy**

- Attending to patients’ social and spiritual needs requires experience and knowledge-based dedication and awareness from healthcare professionals;
- Combining qualitative and quantitative assessment methods helps patients express their needs;
- More research is necessary into the interaction and interconnection of social and spiritual needs specifically and multidimensional needs in general.

## Introduction

Palliative care aims to optimize the quality of life of patients faced with a life-limiting illness and their families by relieving physical, psychosocial, and spiritual suffering.^1^ Both Saunders’ concept of “total pain” and the biopsychosocial-spiritual model of care cover the aim of palliative care and assign a vital role to the social and spiritual dimensions.^1^ Although the social and spiritual dimensions are pivotal in patients’ quality of life and dying,^2,3^ these dimensions are not often adequately assessed in practice.^4–6^ The benefits of assessing these dimensions, however, are widely supported.^7–13^

### Social and spiritual dimensions of quality of life and dying

There is no clear definition of the social dimension of quality of life and dying. Literature shows it encompasses social relationships, which make people happier and give them a sense of identity, companionship, and meaning in life.^3,14^ This dimension also covers emotional support and assisting in practicalities, such as financial help, informational support, and affectionate support.^15^ Connections and relationships give patients a reason to live and enable them to better cope with the reality of dying.^16^ Combining these aspects, sociality can be defined as the dimension of human life related to how people make and maintain relationships with others and that concerns how values, norms, rules, and roles are respected.^14–18^ Life-threatening illnesses can challenge patients’ sociality; limiting their autonomy, independence, and performance status and affecting their social, professional, and family roles as well as changing their bodily appearance.^19^ Consequently, the perception of a limited future makes it more challenging to formulate own goals, expectations, values, and interests concerning one’s social context.^20,21^

In contrast to the social dimension, the spiritual dimension seemingly does have a clear definition. According to the definition adopted by the Spiritual Care Reference Group of the EAPC, spirituality “is the dynamic dimension of human life that relates to the way persons (individual and community) experience, express and/or seek meaning, purpose, and transcendence and the way they connect to the moment, to self, to others, to nature, to the significant and/or the sacred.”^22^ A current review, however, shows definitions of spirituality and the spiritual dimension are, despite the consensus definition, still disputed.^23^ Studies showed that up to 80%–90% of patients had reported spiritual needs.^24,25^ Murray et al. contrasted spiritual with psychosocial needs and defined them as “needs and expectations which humans have to find meaning, purpose, and value in their life.”^4^ For some people, religion is a component of their spirituality, but this may not hold for others. For those who do consider religion meaningful, their level of observance or belief will vary from person to person.^4^

While the social and spiritual dimensions are distinct, the similarities between the linguistic expressions associated with both the social and the spiritual dimension are striking. Social work literature often pays attention to the spiritual dimension by underlining the importance of the existential dimension and the need for collaboration between social workers and spiritual carers to address patients’ needs.^26–28^ The other way around is less the case: literature concerning the spiritual dimension appears to appropriate parts of the social dimension and is more focused on the legitimation of its field.^23^ Concepts like “connection” and “autonomy” are then easily interpreted unilaterally as spiritual, without acknowledging how these concepts relate to the social dimension as well.^23,29^ Studies have mainly focused on patients’ spiritual needs.^24^ Although more and more studies focus on the social dimension of healthcare in general – that is, socioeconomic level, housing, availability –, research concerning patients’ social needs in the palliative phase is staying behind.

### Recurrent themes of patients’ needs

Previous reviews have identified recurrent themes that encompass patients’ needs concerning the spiritual dimension.^30–32^ These themes concern, amongst others, a “need for meaning,” “need for relationships,” “need for control” and “need for independence.” Throughout these reviews and some primary studies, these themes frequently recur, although sometimes phrased differently. Given the recurrence of the same themes throughout studies, it is unlikely for a new study to find patients’ needs concerning the spiritual dimension that do not fit already identified themes. A similar review has also been conducted on patients’ psychosocial needs. Although this review studies psychosocial needs, no definition of the corresponding dimension is provided in the article. This lack of focus could explain why for instance “spiritual distress” is presented as a psychosocial need specific to heart failure patients.^33^ The needs patients express and that are labeled social or spiritual in these reviews show similarities on a linguistic level. Therefore, themes identified for patients’ needs concerning the spiritual dimension are potentially suitable for the social dimension and vice versa.

A thorough review of patients’ social and spiritual needs in the palliative phase of their illness is vital to identifying and understanding patients’ needs, and starting appropriate healthcare interventions, and allocating fitting healthcare providers. Therefore, this mixed-methods systematic review synthesizes the existing evidence on the social and spiritual needs of patients with a life-limiting illness to gain insight into these needs and the distinction between them.

## Methods

### Design

Studies on patients’ social and spiritual needs in the palliative phase of their illness are heterogeneous in terminology and study design.^24^ Since both qualitative and quantitative study designs focus on the same outcomes, being patients’ social and spiritual needs, an integrative mixed-methods design was employed. This design is optimized to fit multiple study designs and types of data.^34^ We employed the Joanna Briggs Institute (JBI) approach to qualitative synthesis or meta-aggregation to synthesize both qualitative and quantitative data.^35^ This method enabled the researchers to assess findings across studies and produce generalizable statements.

Furthermore, performing a literature search within the field of palliative care is challenging due to variations in patient characteristics, diseases, and involved stakeholders, which lead to a broad range of topics.^36–38^ Therefore, we used the Palliative cAre Literature rEview iTeraTive mEthod (PALETTE) framework for developing an appropriate search strategy.^39^ Furthermore, this systematic review was reported following the Preferred Reporting Items for Systematic Reviews and Meta-Analysis (PRISMA) framework.^40^ The protocol of this study was registered in Prospero (registration number: CRD42019133571).^41^ No ethical approval was needed.

### Databases and searches

A preliminary search was conducted in PubMed for studies on patients’ social and spiritual needs in the palliative phase, resulting in the first selected series of studies. By using these studies and consulting information specialists at COCHRANE and specialists within the fields of social work and spiritual care, the terminology employed to describe and study these needs became more transparent, making it possible to gather adequate synonyms, resulting in additional searches. As a result, common needs in the social dimension of healthcare, for example, education, finances and community, turned out to not contribute to the search and were therefore left out. Following the PALETTE method, this process of identifying new studies and adjusting the search string was repeated until the search strategy was validated, meaning it identified all “golden bullets” (see Appendix 1).^39^

The final search was constructed using a Domain Determinant Outcome (DDO) outline (Table 1): D) palliative care, D) the social and spiritual dimensions, O) patients’ needs and the patient perspective. The search was set out on April 28th, 2019, without any limits, and was updated on October 1st, 2020, in five online databases:

**Table 1.** Search strategy PUBMED.

Palliative care	#1	Palliative care[MeSH] OR Terminal care[MeSH]
	#2	Palliative care[Title/Abstract] OR hospice care[Title/Abstract] OR supportive care[Title/Abstract] OR terminal care[Title/Abstract] OR end of life[Title/Abstract] OR advanced cancer[Title/Abstract] OR terminal cancer[Title/Abstract]
	#3	#1 OR #2
Dimension	#4	Religion[MeSH] OR Social behavior[MeSH] OR Psychology[MeSH]
	#5	Religion[Title/Abstract] OR religiosity[Title/Abstract] OR religious[Title/Abstract] OR faith[Title/Abstract] OR spiritual[Title/Abstract] OR spirituality[Title/Abstract] OR existential[Title/Abstract] OR social[Title/Abstract] OR psychosocial[Title/Abstract] OR psychological[Title/Abstract] OR psychology[Title/Abstract] OR spirit[Title/Abstract] OR soul[Title/Abstract] OR meditation[Title/Abstract] OR pray[Title/Abstract] OR rite[Title/Abstract] OR divine[Title/Abstract] or god[Title/Abstract] OR church[Title/Abstract] OR dignity[Title/Abstract] OR hope[Title/Abstract] OR well being[Title/Abstract] OR social support [Title/Abstract] OR family[Title/Abstract] OR network[Title/Abstract] OR family network[Title/Abstract] OR connection[Title/Abstract] OR spouse[Title/Abstract] OR empathy[Title/Abstract]
	#6	#4 OR #5
Needs	#7	Needs assessment[MeSH]
	#8	Need[Title/Abstract] OR issue[Title/Abstract] OR experience[Title/Abstract] OR dilemma[Title/Abstract] OR wish[Title/Abstract] OR demand[Title/Abstract] OR burden[Title/Abstract] OR preference[Title/Abstract] OR unmet[Title/Abstract]
	#9	#7 OR #8
Population	#10	Patients[MeSH] OR attitude to death[MeSH] OR patient satisfaction[MeSH] OR Patient reported outcome measures[MeSH]
	#11	Patient[Title/Abstract] OR patients[Title/Abstract]
	#12	#10 OR #11
Final search	#13	#3 AND #6 AND #9 AND #12

PubMed, EMBASE, CINAHL, Scopus, and PsycInfo. The strategy was validated in PubMed, and minor adjustments were made for use in other databases. The adapted search strategies for the other databases can be found in Appendix 2. Additionally, a manual search of both Google Scholar and reference lists of the included studies was performed by the reviewer (TL) to check the search strategy’s validity.

### Eligibility criteria and study selection

A study was eligible for inclusion if it met the following criteria: (1) the objective of the study was to assess patients’ social and/or spiritual needs; (2) the studied needs were described from a patient’s perspective through either qualitative or quantitative inquiry; (3) the study population needed to consist of patients in the palliative phase of their illness in any healthcare setting; (4) the study was a full report that was published in English, German or Dutch. A “need” was defined as a functional, emotional, social or spiritual issue as perceived by the patient, which requires professional assistance.^42,43^ Preliminary searches showed an increase in study frequency and quality from 2008 onward. Therefore, we excluded studies published before 2008.

Two researchers (TL and EdG) independently screened titles and abstracts to identify relevant studies using Rayyan: a web-based screening program that supports researchers to systematically and methodically select and compare citations independently.^44^ Full texts were retrieved if the abstract did not provide enough information to allow selection or if the study passed the first eligibility screening. Two researchers (TL and EdG) also independently performed full-text screening. When information on eligibility criteria remained unclear after the full-text screening, the first author of the study was contacted by e-mail and requested to send additional information. The researchers resolved any disagreements, and when they did not meet a consensus, this was discussed within the research team.

### Data extraction

Two researchers (TL and EdG) extracted data on the study’s location, research question, study design, participants, setting, data collection method, and outcomes. Based on Cochrane methodology, a purposefully created data extraction form was used to accommodate qualitative and quantitative studies.^45,46^ This form was pilot tested on three qualitative and three quantitative studies to ensure consistency, after which the research team approved them. The data was analyzed using Nvivo 12 software.^20^

### Quality appraisal

The quality of qualitative and quantitative studies was assessed using the JBI Critical Appraisal Checklist for Qualitative Research and the JBI Checklist for Analytical Cross-Sectional Studies.^47^ The checklist for qualitative studies consisted of ten items concerning a study’s congruity, researcher and participants’ position, ethical considerations, and consistency of the conclusions. The quantitative studies consisted of eight items concerning outcome measurement, confounding, and statistical analysis. Two reviewers (TL and EdG) independently appraised the individual components of the checklists by indicating if a study did (+1) or did not (+0) adhere to the quality requirement or whether this was unclear (+0.5). A third reviewer was accessible when a consensus was not reached (ST). A summary score was finally calculated for each study. The quality appraisal did not affect studies’ inclusion since studies with lower appraisal scores could still contain valuable information on patients’ needs.

### Extraction and integration of findings and data-aggregation

Findings from both qualitative and quantitative studies were extracted. A finding was defined as “a theme, category, or metaphor reported by authors of original papers.”^48^ Before aggregating the qualitative and quantitative findings, quantitative data had to be transformed or qualitized into a compatible format. The numerical values of standardized questionnaires were provided with a qualitative label “present” or “not present” in the studied population, comparable to quantitative dichotomization.^49,50^ This leveled out any differences between qualitative and quantitative studies, no longer warranting separate analyses and syntheses of the findings.^50^ This approach fitted this review since both qualitative and quantitative studies addressed the same outcome and research questions.^34^ Findings in both qualitative and quantitative studies were assumed to complement one another.^51^

The JBI method of meta-aggregation was then used to extract findings, categorize these findings, and finally synthesize them. Three levels of evidence exist for these findings: (1) unequivocal, (2) credible, and (3) unsupported.^52^ Unequivocal evidence is supported by citations that were not open for challenge. Credible evidence was supported by citations that were open for interpretation. Unsupported findings were not supported by any citations.^53^

The findings and categories were aggregated based on similarity in meaning. Two researchers (TL & EdG) studied all findings before categorizing them by theme. A consensus on the categories was sought within the research team, after which the findings were synthesized. These synthesized findings were used as a reflection on patients’ needs.

## Results

### Search and selection strategy

**Figure 1.**
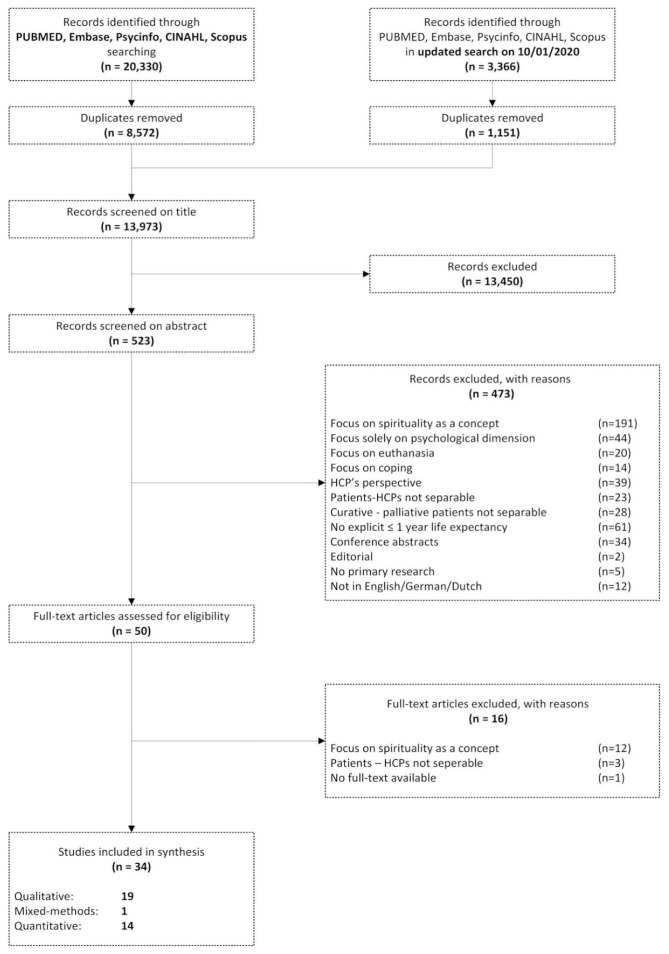
PRISMA flowchart.

The final, updated search yielded 13,973 studies after the removal of duplicates. After title and abstract screening, 50 full texts were assessed for eligibility. This review finally included 34 studies (see Figure 1).

### Characteristics of the included studies

In total, 19 qualitative studies, 1 mixed-method study, and 14 quantitative studies were included, the characteristics of which are presented in Table 2. These studies represent 4465 patients, 2544 (57%) women.

**Table 2.** Study characteristics.

Qualitative studies						
Author/year/country	Study type	Setting	Sample	Method of data collection	Outcome	Quality out of max. 10^c^
Ling Lee and Ramaswamy (2020) Singapore	Phenomenological inquiry	Home	Size: 25	Interview study	Participants’ experience of living with advanced cancer.	6,5/10
			Diagnosis: Cancer			
			Age: 58–76			
			Female: 54.5%			
Lee Mortensen et al. (2018)^54^ Denmark	Generic qualitative study	Hospital	Size: 18	Focus group study	Health-related quality of life and support needs.	9,5/10
			Diagnosis: Cancer			
			Age: 41–72			
			Female: 100%			
Ross and Austin (2015)^55^ United Kingdom	Generic qualitative study	Hospital	Size: 16	Interview study	Spiritual needs and spiritual support preferences and guidelines.	9/10
			Diagnosis: Cardio			
			Age: 60–84			
			Female: 43.8%			
Hatamipour et al. (2015)^56^ Iran	Generic qualitative study	Hospital	Size: 18	Interview study	Spiritual needs.	9/10
			Diagnosis: Cancer			
			Age: 22–72			
			Female: 50%			
O’Connor (2014)^57^ Australia	Generic qualitative study	Home and hospice	Size: 8	Interview study	Experiences.	9/10
			Diagnosis: Cancer			
			Age: 56–86			
			Female: 50%			
Schroedl et al. (2014)^58^ United States	Generic qualitative study	Hospital	Size: 20	Interview study	Unmet healthcare needs.	8/10
			Diagnosis: COPD			
			Age: 52–83			
			Female: 55%			
Simha et al. (2013)^59^ India	Generic qualitative study	Hospice	Size: 10	Interview study	Spiritual concerns.	8,5/10
			Diagnosis: Cancer			
			Age: 38–70			
			Female: 70%			
Bajwah et al. (2013)^60^ United Kingdom	Generic qualitative study	Hospital	Size: 8	Interview study	Palliative care needs.	10/10
			Diagnosis: Lung disease			
			Age: 56–81			
			Female: 37.5%			
Elsner et al. (2012)^61^ India	Generic qualitative study	Palliative Care Clinic	Size: 37	Interview study	Psychosocial and spiritual problems.	9/10
			Diagnosis: Cancer, kidney			
			Age: 56.5			
			Female: 59.5%			
Dehghan et al. (2012)^62^ United Kingdom	Generic qualitative study	Hospital	Size: 3	Interview study	Palliative care needs and care experiences.	9/10
			Diagnosis: Cancer			
			Age: 20–52			
			Female: 66.7%			
Qualitative studies						
Author/year/country	Study type	Setting	Sample	Method of data collection	Outcome	Quality out of max. 10^c^
Chang et al. (2012)^63^ United States	Generic qualitative study	Hospital	Size: 17	Interview study	Spiritual needs.	8,5/10
			Diagnosis: NR			
			Age: 58–94			
			Female: 0%			
Volker and Wu (2011)^64^ United States	Generic qualitative study	Urban and rural site	Size: 20	Interview study	The meaning of control and control preferences.	9/10
			Disease: Cancer			
			Age: 34–87			
			Female: 50%			
Strohbuecker et al. (2011)^65^ Germany	Generic qualitative study	Nursing home	Size: 9	Interview study	Palliative care needs.	9/10
			Diagnosis: chronic			
			Age: 70–100			
			Female: 77.8%			
Hsiao et al. (2011)^66^ Taiwan	Generic qualitative study	Hospital	Size: 33	Interview study	Spiritual needs.	9/10
			Diagnosis: Cancer			
			Age: 51.92 (SD 10.2)			
			Female: 48%			
Nixon and Narayanasamy (2010)^67^ United Kingdom	Generic qualitative study	Outpatient clinic	Size: 21	Questionnaire study	Spiritual needs.	9/10
			Diagnosis: Cancer			
			Age: 18–69			
			Female: 50%			
Shih et al. (2009)^68^ Taiwan	Generic qualitative study	Hospital	Size: 35	Interview study	Constitutive patterns, spiritual needs, and professional actions.	9/10
			Diagnosis: Cancer			
			Age: 75.36 (SD 3.41)			
			Female: 46%			
Wijk and Grimby (2008)^69^ Sweden	Generic qualitative study	Hospital	Size: 30	Interview study	Needs at the end of life.	9/10
			Diagnosis: Cancer			
			Age: 79			
			Female: 50%			
Shah et al. (2008)^70^ United States	Generic qualitative study	Hospital	Size: 226	Content analysis	Concerns.	8,5/10
			Diagnosis: multiple			
			Age: 65.08 (SD 14.73)			
			Female: 50%			
Aoun et al. (2008)^71^ Australia	Generic qualitative study	Home	Size: 11	Interview study	Supportive care needs.	9/10
			Diagnosis: Cancer			
			Age: 73.6 (SD 11.5)			
			Female: 72.7%			
Mixed methods studies						
Author/year/country	Study type	Setting	Sample	Method of data collection	Outcome	Quality out of max. 10 and 8^d^
Egan et al. (2017)^72^ New Zealand	Mixed methods	Hospice/hospital	Interview/Survey^a^	Interviews and The Spirituality in New Zealand Hospice/Palliative Care Survey	Spiritual beliefs, practices, and needs.	8/10 and 7/8
			Size: 24/141			
			Disease: Cancer			
			Age: ?e/67 (SD 10)			
			Female: ?/55%			
Quantitative studies						
Author/year/country	Study type	Setting	Sample	Instrument for of data collection	Outcome	Quality out of max. 8^c^
Yun et al. (2018)^73^ South Korea	Cross-sectional study	Hospital	Size: 1001	Purposeful created instrument	Components of a good death.	7,5/8
			Disease: Cancer			
			Age: <40–>50			
			Female: 60.9%			
Astrow et al. (2018)^74^ United States	Observational study	Outpatient site	Size: 727	The Spiritual Needs Assessment for Patients (SNAP)	The dimension of spiritual need.	8/8
			Disease: Cancer			
			Age: 59 (SD 16.8)			
			Female: 67.8%			
Delgado-Guay et al. (2016)^75^ United States	Randomized controlled trial	Palliative Care Unit	Size: 100	Purposefully created Go Wish card game List of wishes/statements	Wishes at the end of life.	7/8
			Disease: Cancer			
			Age: 27–83			
			Female: 60%			
Uitdehaag et al. (2015)^76^ The Netherlands	Cross-sectional study	Outpatient clinic	Size: 56	Problems and Needs in Palliative Care questionnaire (PNPC)^77^, EORTC-QLQ-OES18^78,79^ and EORTC QLQ-PAN26^80^	Problems and needs.	7/8
			Disease: Cancer			
			Age: 65 (SD 11.8)			
			Female: 21.1 %			
Effendy et al. (2015)^81^ The Netherlands/Indonesia	Cross-sectional study	Hospital	Indonesia/Netherlands^b^	The Problems and Needs in Palliative Care questionnaire (PNPC)^77^	Problems and unmet needs.	8/8
			Size: 180/94			
			Disease: Cancer			
			Age: 21–77/30–87			
			Female: 73.9%/70.2%			
Dedeli et al. (2015)^82^ Turkey	Cross-sectional study	Hospital	Size: 230	Purposefully created instrument and Turkish adaptation of Patients Spiritual Needs Assessment Scale (PSNAS)^83^	Spiritual needs.	8/8
			Disease: Cancer			
			Age: 55.3 (SD 15.8)			
			Female: 52.2%			
Vilalta et al. (2014)^84^ Spain	Observational study	Palliative Care Unit	Size: 50	Purposefully created questionnaire	Spiritual Needs.	7,5/8
			Disease: Cancer			
			Age: 48% > 60			
			Female: 38%			
Quantitative studies						
Author/year/country	Study type	Setting	Sample	Instrument for of data collection	Outcome	Quality out of max. 8^c^
Höcker et al. (2014)^85^ Germany	Cross-sectional study	Radiation clinic	Size: 285	Spiritual Needs Questionnaire (SpNQ)^86^	Spiritual needs.	8/8
			Disease: Cancer			
			Age: 18–83			
			Female: 49.8			
Pearce et al. (2012)^87^ United States	Cross-sectional study	Oncology unit	Size: 143	Adaptation of Functional Assessment of Chronic Illness Therapy – Spiritual Wellbeing Scale (FACIT-Sp)^88^	Unmet spiritual care needs.	8/8
			Disease: Cancer			
			Age: 58.6 (SD 14.2)			
			Female: 49.3%			
Fitch (2012)^89^ Canada	Cross-sectional study	Radiation clinic	Size: 69	Adaptation of Supportive Care Needs Survey — Radiation (SCNS-R)^90^	Supportive care needs.	7,5/8
			Disease: Cancer			
			Age: 65 (35–84)			
			Female: 49.3%			
Ugalde et al. (2011)^91^ Australia	Cross-sectional study	Hospital	Size: 108	Needs Assessment for Advanced Lung Cancer Patients (NA-ALCP)^92^	Unmet needs.	7/8
			Disease: Cancer			
			Age: 39–83			
			Female: 40%			
Ben Natan et al. (2010)^93^ Israel	Cross-sectional study	Geriatric center	Size: 73	Purposefully created questionnaire	End-of-life needs.	6,5/8
			Disease: ?			
			Age: ?			
			Female: 66.6%			
Rainbird et al. (2009)^94^ Australia	Cross-sectional study	Hospital	Size: 246	Needs Assessment for Advanced Cancer Patients questionnaire (NA-ACP)^95^	Unmet needs.	6.5/8
			Disease: Cancer			
			Age: 61 (SD 11.9)			
			Female: 53%			
Cheng et al. (2008)^96^ Taiwan	Cross-sectional study	Palliative care unit	Size: 366	The good death scale^97^ and the audit scale for good death services^98^	Factors related to a good death.	6,5/8
			Disease: Cancer			
			Age: 65 (SD 16.49)			
			Female: 50%			

a This mixed-methods study presents results on two studies separately – for example, populations are also presented separately.

b Cross-sectional data were compared for two populations and presented separately.

c This score is compiled out of the quality appraisal that can be seen in Tables 3–5.

d The quality of the qualitative and quantitative arms of this mixed-methods has been assessed separately.

e When a variable is unknown, this is presented as “?”.

The qualitative studies report on the results of 565 patients, 292 (52%) women, with a median of 18 participants per study. 13 of the 19 studies focused on cancer as the primary diagnosis,^54,56,57,59,61,62,64,66–69,71,99^ others focused on heart disease (*n* = 1),^55^ lung disease (*n* = 2),^58,60^ and multiple (chronic) diseases (*n* = 2).^65,70^ For one study the diagnosis was not reported.^63^ Data was collected through interviews (*n* = 16),^55–66,68,69,71,99^ a focus group (*n* = 1),^54^ a qualitative questionnaire (*n* = 1),^67^ and patient records (*n* = 1).^70^ Populations from these studies mostly originated from Europe (*n* = 7),^54,55,60,62,65,67,69^ Asia (*n* = 5),^59,61,66,68,99^ and the United States (*n* = 4),^58,63,64,70^ followed by Oceania (*n* = 2),^57,71^ and the Middle East (*n* = 1).^56^

The mixed-methods study included 165 patients, 90 (54%) women, divided over a qualitative and a quantitative arm.^72^ Data was collected through interviews and a survey on patients who have cancer.

The quantitative studies included 3728 patients, 2162 (58%) women, with a median of 165 participants per study. All quantitative studies included patients with cancer,^73–76,81,82,84,85,87,89,91,94,96^ except one in which the type of disease is unclear.^93^ Study designs were predominantly cross-sectional survey studies (*n* = 11).^73,76,81,82,85,87,89,91,93,94,96^ Other designs were an observational study (*n* = 2),^74,84^ and a randomized controlled trial (*n* = 1).^75^ Origination of the populations from quantitative studies was: United States (*n* = 4),^74,75,87,89^ Europe (*n* = 4),^76,81,84,85^ Oceania (*n* = 2),^91,94^ Middle East (*n* = 2),^82,93^ and Asia (*n* = 2).^73,96^

Of the included studies, European and Asian studies predominantly had a qualitative study design: respectively 6/10 and 6/8. Oceanian and Middle Eastern studies predominantly had a quantitative design, respectively, 3/5 and 2/3. Study designs from studies in the United States were divided fifty-fifty. Results showed that not all needs are found equally by qualitative and quantitative studies.

### Quality appraisal

Tables 3, 4, and 5 show the quality appraisal of the included studies.

**Table 3.** Quality appraisal of qualitative studies.

	Yes (n total=19)	Lee 2020	Mortensen 2018	Ross 2015	Hatamipour 2015	O’Connor 2014	Schroedl 2014	Simha 2013	Bajwah 2013	Elsner 2012	Dehghan 2012	Chang 2012	Volker 2011	Strohbuecker 2011	Hsiao 2011	Nixon 2010	Shih 2009	Wijk 2008	Shah 2008	Aoun 2008
Is there congruity between the stated philosophical perspective and the research methodology?	18	N	Y	Y	Y	Y	Y	Y	Y	Y	Y	Y	Y	Y	Y	Y	Y	Y	Y	Y
Is there congruity between the research methodology and the research question or objectives?	19	Y	Y	Y	Y	Y	Y	Y	Y	Y	Y	Y	Y	Y	Y	Y	Y	Y	Y	Y
Is there congruity between the research methodology and the methods used to collect data?	18	U	Y	Y	Y	Y	Y	Y	Y	Y	Y	Y	Y	Y	Y	Y	Y	Y	Y	Y
Is there congruity between the research methodology and the representation and analysis of data?	18	N	Y	Y	Y	Y	Y	Y	Y	Y	Y	Y	Y	Y	Y	Y	Y	Y	Y	Y
Is there congruity between the research methodology and the interpretation of results?	19	Y	Y	Y	Y	Y	Y	Y	Y	Y	Y	Y	Y	Y	Y	Y	Y	Y	Y	Y
Is there a statement locating the researcher culturally or theoretically?	16	Y	Y	Y	Y	Y	Y	U	Y	Y	Y	Y	Y	Y	Y	N	Y	Y	U	Y
Is the influence of the researcher on the research, and vice versa, addressed?	2	N	U	N	N	N	N	N	Y	N	N	N	N	N	N	Y	N	N	N	N
Are participants, and their voices, adequately represented?	19	Y	Y	Y	Y	Y	Y	Y	Y	Y	Y	Y	Y	Y	Y	Y	Y	Y	Y	Y
Is the research ethical according to current criteria, and is there evidence of ethical approval?	17	Y	Y	Y	Y	Y	N	Y	Y	Y	Y	U	Y	Y	Y	Y	Y	Y	Y	Y
Do the conclusions drawn in the research report flow from the analysis or interpretation of the data?	19	Y	Y	Y	Y	Y	Y	Y	Y	Y	Y	Y	Y	Y	Y	Y	Y	Y	Y	Y
**Total out of 10**		6.5	9.5	9.0	9.0	9.0	8.0	8.5	10	9.0	9.0	8.5	9.0	9.0	9.0	9.0	9.0	9.0	8.5	9.0

Y : yes; N : no; U : unclear.

**Table 4.** Quality appraisal of mixed-method study.

	Egan 2017 (1)^a^		Egan 2017 (2)^b^
Is there congruity between the stated philosophical perspective and the research methodology?	Y	Were the criteria for inclusion in the sample clearly defined?	U
Is there congruity between the research methodology and the research question or objectives?	Y	Were the study subjects and the setting described in detail?	Y
Is there congruity between the research methodology and the methods used to collect data?	Y	Was the exposure measured validly and reliably?	Y
Is there congruity between the research methodology and the representation and analysis of data?	Y	Were objective, standard criteria used for measurement of the condition?	Y
Is there congruity between the research methodology and the interpretation of results?	Y	Were confounding factors identified?	Y
Is there a statement locating the researcher culturally or theoretically?	U	Were strategies to deal with confounding factors stated?	U
Is the influence of the researcher on the research, and vice versa, addressed?	N	Were the outcomes measured validly and reliably?	Y
Are participants, and their voices, adequately represented?	U	Was an appropriate statistical analysis used?	Y
Is the research ethical according to current criteria, and is there evidence of ethical approval?	Y		
Do the conclusions drawn in the research report flow from the analysis or interpretation of the data?	Y		
**Total out of 10**	8.0	**Total out of 8**	7.0

Y : yes; N : no; U : unclear.

a Egan 2017 (1) considers the qualitative arm of the study.

b Egan 2017 (2) considers the quantitative arm of the study.

**Table 5.** Quality appraisal of quantitative studies.

	Yes (n total =14)	Yun 2018	Astrow 2018	Delgado 2016	Uitdehaag 2015	Effendy 2015	Dedeli 2015	Vilalta 2014	Höcker 2014	Pearce 2012	Fitch 2012	Ugalde 2012	Ben Natan 2010	Rainbird 2009	Cheng 2008
Were the criteria for inclusion in the sample clearly defined?	12	Y	Y	U	U	Y	Y	Y	Y	Y	Y	Y	Y	Y	Y
Were the study subjects and the setting described in detail?	14	Y	Y	Y	Y	Y	Y	Y	Y	Y	Y	Y	Y	Y	Y
Was the exposure measured validly and reliably?	10	U	Y	Y	Y	Y	Y	Y	Y	Y	Y	Y	U	U	U
Were objective, standard criteria used for measurement of the condition?	14	Y	Y	Y	Y	Y	Y	Y	Y	Y	Y	Y	Y	Y	Y
Were confounding factors identified?	10	Y	Y	Y	Y	Y	Y	Y	Y	Y	Y	U	U	U	U
Were strategies to deal with confounding factors stated?	6	Y	Y	U	U	Y	Y	U	Y	Y	U	U	U	U	U
Were the outcomes measured validly and reliably?	14	Y	Y	Y	Y	Y	Y	Y	Y	Y	Y	Y	Y	Y	Y
Was an appropriate statistical analysis used?	14	Y	Y	Y	Y	Y	Y	Y	Y	Y	Y	Y	Y	Y	Y
**Total score out of 8**		7,5	8	7	7	8	8	7,5	8	8	7,5	7	6,5	6,5	6,5

Y : yes; N : no; U : unclear.

Fourteen of the nineteen qualitative studies achieved at least 9/10 quality criteria. The main weakness across these studies was a lack of reflexivity, with only two of the nineteen qualitative studies achieving this criterion. As a result, the influence of the researchers on data collection and analysis is not apparent.

In the mixed-methods study, data on the qualitative arm was not reported in as much detail as was the data on the quantitative arm.

Of the quantitative studies, eleven of the fourteen studies achieved at least 7/8 quality criteria. The main weakness across these studies was a lack of strategies to control for confounding, with only seven of the fifteen studies achieving this criterion.

A summary score of the quality assessment is presented in Table 2. The quality of the studies was not of influence on the aggregation.

### Transformation of quantitative data

Quantitative studies all used surveys to identify the prevalence of social and spiritual needs through prespecified items. These items were organized thematically or divided into subcategories: “social needs,” “spiritual needs,” “religious needs,” or even more specific “reassurance,” and “thoughts about end of life.” In this study, the themes/subcategories in the surveys were regarded as equal to the themes that resulted from thematic analyses in qualitative studies. Studies focusing on patients’ needs in a more general fashion only thematized their items to their corresponding dimensions, for example, “social needs,” “spiritual needs.” In these instances, the individual items of the survey were regarded as distinct themes. Themes and items from surveys were only reported as findings in the synthesis when patients reported them as prevalent.

### Synthesis and meta-aggregation of findings

We developed five synthesized findings from 18 categories based on 243 unique findings: 84 qualitative and 159 quantitative findings. Table 6 shows the synthesized findings, categories, and findings. For qualitative findings, the level of evidence of these findings is reported: (1) unequivocal, (2) credible, and (3) unsupported. Since quantitative studies report on the prevalence of needs and provide no further illustrations, no level of evidence is reported. When a quantitative 5 finding is prevalent, it is included in the meta-aggregation.

**Table 6.** Synthesized findings, categories, findings, and their level of evidence.

Synthesized findings	Categories	Findings	
**Being autonomous**	Need to be treated as a person	**Qualitative findings and illustrations**	
		Being recognized as a person (C)	“To become acquainted with one or two nurses.”^65^
			“I really like it when they come to see me in the evening, after the news and ask me if everything is okay or if I need anything [. . .] And when they give me a backrub. It just takes a few minutes.”^65^
		The need to maintain physical and spiritual integrity (C)	“Since we know little about death, most of us have a lot of questions. Health professionals’ support, which is demonstrated through patiently answering our questions, is invaluable.”^68^
			“Please do not let me look ugly; I need to be good looking when I enter the afterlife.”^68^
		Normal behavior (U)	“I have this disease, and I’m living my life, studying, and maybe ahead of apparently healthy people, scientifically and mentally. That’s why I don’t like them pity me; I detest pity. I like them to pray for me, and come and visit, but not because they pity me.”^56^
		Concept of loss (C)	“Yes, I feel bad, but not that bad. Enough, My body is a waste, feel repulsive (repeated twice). A waste, for my children, for my parents, for people like you who serve others, a waste.”^59^
		Loss of jobs & social prestige (C)	“I have debts of Rs 50,000 due to my daughter’s marriage. . . Before my death, she has to get married, so I arranged a marriage for her, and she got married. I borrowed money from a family, but they want their money back because their daughter will be getting married shortly.”^61^
		**Quantitative findings** ^a^	
		To be treated the way I want	To keep my sense of humor
		To have a human touch	Be called by name
		To maintain my dignity	Take opinion seriously
		Respect for autonomy	To be treated as a person, not just another case
		To have a doctor who knows me as a whole person	Recognition as a person until the end of life
	Need to be in control	**Qualitative findings and illustrations**	
		Maintain independence (U)	“I’m fairly independent, and I’ve sort of tried since I got the illness to keep things as normal as I could.”^57^
			“I’m one of those people who would never interfere in someone else’s life and tell them what to do, and I really don’t like anyone doing it to me either.”^57^
		Balancing independence and the need for assistance (U)	“I’m ﬁercely independent, and I’d rather crawl on my hands and knees to do something, rather than have people do it for me. But sometimes, you have to have a bit of care.. [A care aide]. Gives me all the conﬁdence in the world.”^57^
		Reliance on others and the change in relationships (U)	“You never think that you are going to get in a position where you um you can’t look after um everybody else.”^60^
			“We’re not intimate at the moment (higher pitch voice) because I find it (. . .) we don’t even talk about it, we just sort of blank it out because I just don’t have the will or the energy.”^60^
		It is mine to keep together (U)	“I want to be able to have control or say about my illness, whether I think I should take chemo [chemotherapy] or not. The doctors tried to talk me out of it [her decision to stop treatment], and it’s just like, it’s MY body.”^64^
			“Me not taking chemo, that’s control.”^64^
			“I got a hand in what is going on. I got to know what’s in the bottle; I got to know how long it’s going to take. I got to know what tests they are doing and why they are doing that test. Why are they doing this? What are they looking for?”^64^
		God controls our lives (U)	“Actually, I don’t think you can have control. I think you are given an opportunity, because you can’t control something you can’t heal, and you can’t control something you can’t make. Only the good Lord can do that. But you do to other people, help other people.”^64^
			“The Lord knew I had cancer even before I did. Already knew what I had before the storms [Hurricane Katrina]. And He wanted me to be in a place [Texas] where He knew I could be taken care of.”^64^
		Having a choice and being in control (U)	“Well, that, if patients want it, they [nurses] would not just do it in a stereotyped way—but would ask you if you would like a bath with a washcloth, or take a shower, or a bath in the tub today.”^65^
			“Well, I am not very demanding. But I pay attention and I need to see what they give to me. In the beginning, well, of course, they did not know me—they just poured it [medicine] into my mouth.”^65^
		**Quantitative findings** ^a^	
		Being dependent on others	Experiencing loss of control over one’s body
		Loss of control over one’s life	Experiencing loss of control over one’s life
		Not being connected to machines	To be treated as a person, not just another case
		Experiencing difficulties in asking for help	To be treated the way I want
		Reluctance giving tasks out of hands	
	Need to continue life as usual	**Qualitative findings and illustrations**	
		The extent of physical and psychosocial needs (C)	“I’m breathless, always breathless.”^60^
		Impact of the disease on social activities (U)	“I can’t go anywhere [. . .] . . . I don’t [really] have a life I’m sitting indoors everyday . . . I used to be meet friends and have coffee and it [would] give you a bit of life back.”^60^
		Plans for the future/sense of normality (U)	“I just wanted to get on with getting it sorted out, not worrying other people too much, and to get back to work and normality.”^67^
		Living day by day (U)	“This is my life now, nothing I can do….Now that I am sick, nothing is important.”^99^
		**Quantitative findings** ^a^	
		Coping with frustration of not being able to do things you used to	Difficulties in continuing usual activities/social activities
		Changes in usual routine and lifestyle	Difficulties in employment or following study
		Frustrations because inability to do as much as before	Difficulties in doing heavy/personal transportation/shopping/preparing meals or cooking/rising, walking, climbing stairs/doing light housework/body care, washing, dressing, or toilet
	Need to deal with financial concerns	**Qualitative findings and illustrations**	
		Socio-economical clarification (C)	“And then you get to sit there afterwards and think ‘God, yes, I do actually feel awful and there are all these things I can’t do’. The option of staying on sickness benefit [with a flex job in this case] rather than receiving disability pension is really good.”^54^
		Financial problems (Uns)	
		Social needs (C)	“My father is a farmer. He can’t manage all the money for my treatment. I have three sisters, two older and one younger. . . I used to earn a lot of money, used to take care of my home. . . I won’t live, what will happen to my father, my mother [crying]?”^62^
		**Quantitative findings** ^a^	
		Finances in order	Extra expenditure because of the disease
		To have my financial affairs in order	Reduced income because of the disease
		Difficulties filling out forms	Difficulties in making arrangements
		Dealing with concerns about financial situation	Difficulties in making life companion acquainted with financial and administrative issues
		Paying the non-medical costs of your illness	Coping with organizing financial situation
	Need to be informed about medical condition	**Qualitative findings and illustrations**	
		Information needs (Uns)	
		Understanding of disease (diagnosis, severity, and prognosis) (U)	“I mean, I had not smoked for 20 some years. It didn’t make any sense to me.”^58^
			‘What can I expect?”^58^
		The fear of knowing more (U)	“If I were to know about my illness I would feel tensed.”^61^
		Need for information (Uns)	
		**Quantitative findings** ^a^	
		To be fully informed about your medical test result as soon as possible	Making ethical decisions about care
		Receiving accurate medical judgments from the medical staff	Lack of information in written
		Being able to have an open discussion with your doctors	Difficulties in remembering what was told
		Getting adequate information from medical staff about side effects and prognosis	To trust my doctor
		Decision-making participation	Need for truth
	Need to be informed about future	**Qualitative findings and illustrations**	
		Concerns about the future (U)	“I’ve got a grandson that wants to become an NBA star. And I told him I’ll live to see him play one game.”^58^
		**Quantitative findings** ^a^	
		Dealing with fears about what is going to happen to you	Difficulties in coping with unpredictability of the future
		Fears about the future	
**Being connected**	Need to maintain relations	**Qualitative findings and illustrations**	
		Love and belonging (U)	“I don’t meet people. The only people I see now is my carers. Otherwise I don’t see a soul. I don’t see anybody. . . Oh very very lonely. Very very lonely.”^55^
		Social support (U)	“My family supports me both physically and mentally, and keeps me spirited. Even though they want to remove my ovary and uterus, everyone reacts as though it’s not a problem. [They say] at most you can’t have a baby, your own health is more important. They support me in every way.”^56^
			“I expect my friends to visit me, and have a heart‑to‑heart. My relative came to visit and made me very happy. That’s the spirit. They could have not cared and not come. Whenever they saw me, they wished me God’s blessing. Well that keeps my spirit high.”^56^
		Being connected to family (U)	“. . .on Sundays, around half past 11 [. . .] when the phone is ringing, I know it is [name of niece]. [. . .] That really makes me happy, that’s lovely. [. . .] And I really can rely on it, oh yes, yes.”^65^
			“If I need them, they’ll be here right away. I’ll never get lost in misery. That’s worth a lot.”^65^
		The need to experience more reciprocal human love (U)	“I recite the Buddha’s name to help me forgive my Daddy since he tortured me and didn’t allow me to receive elementary education because I am a girl. It might have made mistakes and owed him a debt from my last life.”^66^
		Being connected with the world outside (U)	“Being connected to other people. Being connected to people outside and to people here in the nursing home, being in some kind of relationship.”^65^
		Need for connection/loneliness/depression (C)	“Post operation I was down as I couldn’t think what the correct words were and struggled explaining test phrases.”^67^
		Social needs (C)	“. . . to see my brother, never told you that I have a brother, did I? I regret not staying in contact with him during all these years. But I do hear from my son now. It made me happy and moved me. But is it too late now?”^69^
		Feeling closer to d Go (U)	“I have only some emotional problems but I can’t express it. If you are affected by an illness will you get closer to God? You don’t pray when you are safe but I am able to understand God.”^61^
		Being spiritually connected (C)	“I have confidence that there will be a good end and that every step to the end will bring a good result. Well, God, who guides you into it, will help you to pass and lead you out.”^65^
		Social isolation (U)	“You don’t have a social life.”^58^
			“I ﬁnd myself apologizing a lot. I am sorry I cannot do this or that.”^58^
		Strengthening spiritual belief (C)	“When you sincerely want something from God, he gives it. When you go to a shrine with clear conscience, you get results, otherwise you won’t.”^56^
		Loss of social networks (U)	“And I think what it is, ‘We hear that he’s really sick’ and ‘If he gets all horrible and wasted’. Where they’re a bit fearful. When they visit, what they’ll be presented with.”^57^
			“Sometimes I get a bit lonely. The worst time is six o’clock at night, when you come in, shut the door and lock it, and put the lights on.”^55^
		Solitude (U)	“Just needed to accept this on my own.”^67^
		Need for companionship linked to struggle with embarrassment and vulnerability (U)	“Although I feel lonely and scared because of my declining health (during the evening and night in particular), I was embarrassed to ask for my children’s comfort and companionship because I used to be their leader and protector.”^68^
		Marital intimacy (U)	“[It was] better than previously because previously, all the while I was working [long] hours. Sometimes I came back at 10 o’clock. . .they had already slept.”^99^
		Connectedness with friends (U)	“When they come, I am very happy. We talk and I never feel that I am sick….Then about for a month, they never come. They called….sometimes they are scared to come because [I am] sick, so they are scared to disturb me.”^99^
		**Quantitative findings** ^a^	
		Being forsaken by others	To have a nurse I feel comfortable with
		Problems in the contact with (one of) the children	Presence of family
		Problems in contact with family, friends, neighbors, or colleagues	To have my family with me
		Family and friends to be allowed with you in hospital whenever you want	Your relationship with God
		Dealing with maintaining relationships with family members	Becoming closer to a spiritual community
		Being at one with God	Fear of being alone
		Praying	Loneliness
		Continuity of social support	Problems in relationship with life companion
		At peace with God	Difficulties keeping confidence in God or religion
		To have close friends near	A connection with a higher power
		To perform religious or spiritual rituals	Life beyond the individual
		Religious rituals such as chant, prayer, lighting candles	
	Need to care for loved ones or be taken care of by loved ones	**Qualitative findings and illustrations**	
		Family support (U)	“I needed my family with me.”^67^
		The perception of being a burden (U)	“All these days, I’ve lived an active, independent life. I could earn my livelihood. Now, I’m totally incapacitated; I have to depend on others for everything.”^61^
		Relationships (U)	“I don’t want to interfere with my children’s lives. I don’t want to do this to them.”^70^
		Psychological and spiritual needs (C)	“I don’t feel like discussing [my feelings. . .] because then they [family] will be upset. . . if they listen to these things of mine they might get upset. . .”^62^
		Children are participant’s life (C)	“I can feel they are shattered inside because [I am] the mother….So I say my children are brave. So, I’m taking their bravery and I say. ‘I have to face it.’”^99^
		**Quantitative findings** ^a^	
		Capacity to help others	Solace someone
		Worries you have about your family	Concerns about the worries of those close to you
		Dealing with concerns about your family’s fears and worries	Concerns about the ability of those close to you to cope with caring for you
		Not being a burden to my family	Finding it difficult to talk about the disease because of not wanting to burden others
		To have my family prepared for my death	Experiencing little support by others
		To be able to help others	To prevent arguments by making sure my family knows what I want
		Experiencing too little support by others	
	Need to communicate with others	**Qualitative findings and illustrations**	
		Need to talk (U)	“I needed to cry and be allowed to talk about my fears of not seeing my grandchildren ever, of not seeing my sons ever married or settled down. My fear relating to my son who has depression and what might happen to him. My sadness at leaving my husband after 32 years of marriage. I felt I was being hushed when I tried to say these things and that made me more upset. I needed to cry and say them.”^67^
		Communication barriers in families (C)	“I sometimes feel that they might be feeling frustrated because of my condition. They haven’t shown anything openly so far – they are bearing everything patiently – maybe also because of the way I have treated them all these years.”^61^
		Communicate with God (C)	“One has more faith in this period. I did have faith before, but I feel closer to God now.”^56^
		Prayer (U)	“Besides treatments, the only cure is prayer. The doctor may diagnose, but with prayer his diagnosis may change easily. Perhaps my prayer is above his diagnosis, and surely that’s so.”^56^
			“Prayer calmed me, and I felt closer to God; I forget my illness. It gave me more will. I felt calmer, the more I prayed, and forgot my illness.”^56^
		Spiritual needs (C)	“Sometimes it helps just to talk to someone. . . . to get out of your own mind and just to talk.”^63^
			“I am 77 so I can see the age is the cliff coming up. And wonder what on the other side. …. I’d rather solve it (the question) myself, find my own answers.”^63^
			“My spiritual needs at this stage in my life [are] to be in as strong and as spiritual a setting as I can be in surrounded by my Christian brothers and sisters and doctors too and that’s being provided at the VA.”^63^
		Talking to God (C)	“Yes, this is all God’s will. He is the one who gave me the illness. He is the one who can heal me. That is all I hope for.”^99^
		**Quantitative findings** ^a^	
		Talking to other people about cancer	Conversation with cleric about the meaning of death
		Sharing your thoughts and feelings with people close to you	To be able to talk about what death means
		Dealing with the reactions by your family and/or friends to your illness	To be able to talk about what scares me
		Being able to express feelings with friends and/or family	Difficulties in talking about the disease with the life companion
		To have someone who will listen to me	Difficulties in finding someone to talk to (confidant)
		Need for religious expression	
	Need to get in touch with others	**Qualitative findings and illustrations**	
		None.	
		**Quantitative findings** ^a^	
		Visits from a hospital chaplain	Visits from clergy of own faith community
		To meet with clergy or chaplain	Getting in touch with other patients with similar disease
		Difficulties being available to others	Visits from fellow members of your faith community
		Turn to a higher presence	
**Finding and having meaning**	Need to accept and find meaning in situation and/or illness	**Qualitative findings and illustrations**	
		Acceptance of one’s situation (C)	“I had faith in God. I am not worried as I leave everything to Him.”^59^
			“Karma is what I am experiencing now, Yes, I may have sinned. I do believe in karma. I feel very bad that such a thing has happened to me.”^59^
		Accepting reality (U)	“I think of the disease as part of my body and I have gotten so used to it. It fights me and me, too.”^56^
			“Why should have God wished me this disaster, well what God gives can’t be disaster, but why me. I feel he always chooses the best, and that calms me.”^56^
		Religious needs (U)	“Whole thing has awakened in me stirred in me religious beliefs. Whole reappraisal of life.”^67^
		Seeking meaning (U)	“When I think about my disease, the question arises, why me? And the answer comes to me. I feel I got ill because I was better in some things than others, or else God wants to test me, to see how thankful we are in every circumstance, he wants to see if we are up to it or not.”^56^
		Spirituality (U)	“I’ve started to ponder. Ask myself if I’m religious. I’ve never thought about those things before, but I do now.”^69^
		Changing meaning of life (U)	“Sometimes I talk with my disease. You have come, but rest assured I’ll beat you; I won’t let you win. I fight you twice as hard as you fight me. I really feel there is a tenant living in my body that should soon leave.”^56^
		Passive acceptance of their situation (U)	“I had faith in God. I am not worried as I leave everything to Him.”^59^
			“Karma is what I am experiencing now, Yes, I may have sinned. I do believe in karma. I feel very bad that such a thing has happened to me.”^59^
		**Quantitative findings** ^a^	
		Meaning and purpose	Difficulties accepting the disease
		Finding meaning in experience	Confusion about why this has happened to you
		Coping with suffering	Finding meaning
		Finding meaning in illness and/or suffering	Make sense of why this happened to you
	Need to have had a meaningful life	**Qualitative findings and illustrations**	
		Thoughts about meaning of life (U)	“I felt guilty that I had not done enough in my life to prepare for this.”^67^
		Dying without regret (U)	“I ask myself, ‘What things can I leave behind that will be remembered by others?’ The more positive the answers, the more peace and the less regret I have.”^68^
		The need to fulfill the meanings of life and preserve one’s dignity (U)	“Sometimes I want to die; but, I owed others a lot of money. I should return the money then die innocently since I don’t want to rebirth in the world again.”^66^
		Ascertaining a sustained being in the world (U)	“I asked my oldest son to demonstrate his filial piety by compliance with my unfulfilled duties, such as taking over my role of caring for his siblings and deal with family issues.”^68^
		Meaning and purpose (U)	“I feel that my usefulness on earth is ﬁnished now. I’m neither use nor ornament now really.”^55^
		Searching for belonging in the future world by attaining a firm sense of religious affiliation (U)	“Living is a daily battle for an old dying person; I simply can’t stay independent like I used to be. Religious support has been like cool drink of water and a crutch to help me on my daily walk through the desert.”^68^
		Having a prosperous end (C)	“I wish it all ends well when I pray. I’m not afraid of people. All I want is not to burden myself on my kids, not to suffer, not to need their help, so they don’t tire. I don’t want to be a bother for anyone.”^56^
		**Quantitative findings** ^a^	
		To perform religious or spiritual rituals	Affirmation of one’s past life
		Plunge into beauty of nature	Not experiencing pleasure anymore
		Divine	Feel life was meaningful
		Appreciating beauty	To feel that my life is complete
		Feelings of futility	Find a meaning for existence
		The meaning and purpose of human life	Someone bring you spiritual texts
		To remember personal accomplishments	
	Need for forgiveness	**Qualitative findings and illustrations**	
		Begging forgiveness (U)	“I always say, you gave me the disease, and I don’t know when I may go, but I want to go forgiven.”^56^
		**Quantitative findings** ^a^	
		Freedom from blame and guilt, and forgiving others	Reconciliation and forgiveness
		Finding forgiveness	Wanted to forgive yourself or others
	Need for completion	**Qualitative findings and illustrations**	
		None.	
		**Quantitative findings** ^a^	
		Setting new priorities in life	Fulfillment of last wish
		Making the most of your time	Resolve unfinished business
		Dissolve open aspects in life	To take care of unfinished business with family and friends
		Resolving old disputes	
**Having a positive outlook**	Need for hope	**Qualitative findings and illustrations**	
		Hope and coping (U)	“It’s a bit of a downer really, I haven’t seen this doctor before. . .I said well it makes you feel better (referring to doing gentle exercise). . .he said ‘well why do it, there’s nothing there to build on, you’ve got nothing at all’. I didn’t need that at the time you know. I needed somebody to build me back up.”^55^
		Hopefulness (U)	“When you know in your heart and have hope in God, surely God will help, your spirit will be high and you can defend yourself.”^56^
		Faith in God (U)	“With all the possibilities today, when one falls ill, one doesn’t think about dying. One thinks about the doctors and specialists. That’s God’s will. He gives the disease and the cure.”^56^
		The need to foster hope for survival and obtain a peaceful mind-set (C)	“Truth-telling helps patients learn better about their cancer status and have a hope for survival. Then, they’ll be less frightened.”^66^
		Reassurance (U)	“Needed reassurances – about survival, to deal with inner panic and disappointment and fears.”^67^
		Hope (C)	“I don’t want people to think I’m sick, not moving, not doing anything, and yet I am bothering them with all my unnecessary complains when they are also busy with their own job…. I don’t think I like it [the situation]. . . I don’t want to overstay my worldly time.”^99^
		Hopelessness (U)	“Sometimes lying down, I think to myself, ‘Having done so much, what did I get in return?’ Because of this, that’s why you see, I don’t really bother much about caring for the kids [grandchildren]. I just tell them to go and do what they want….I just give up hope.”^99^
		**Quantitative findings** ^a^	
		Need for hope	Difficulties seeing positive aspects of the situation
		Hospital staff to convey sense of hope	Keeping a positive outlook
		Finding hope	Positive/gratitude/hope/peace
	Need for peace	**Qualitative findings and illustrations**	
		Seeking inner peace (U)	“Sometimes I like to be on my own, but not for just thinking, it is good for prayers and reading the Quran. When I’m alone, I pray better and do my work better. Not that I sit down and ponder, but I like to be alone for communication with God.”^56^
			“When I’m under immense pressure, I put on my tracksuit and go out. To satisfy myself and snap out, I read a book. That’s my fate.”^56^
		Active behaviors (U)	“I have looked after my responsibilities toward my family well. I have not committed any mistake. Yes, one is selfish about ones husband and children.”^59^
			“I do pooja to pay off the (karmic) debts of my previous birth. I have not missed doing pooja. It has been useful for me. The mind is satisfied and peaceful.”^59^
		Meaning of prayers (U)	“After going to the prayer I am getting relief and also I get a good sleep. . .When I have pain I pray to God and after the prayer I don’t know where the pain is gone.”^61^
		**Quantitative findings** ^a^	
		Personal meditation or prayer practices	Dwell at a place of quietness and peace
		Overcoming fears	Need for freedom and to be free
		Difficulties in relaxing	Relaxation or stress management
		Find peace of mind	
**Dealing with dying and death**	Need to deal with dying and death	**Qualitative findings and illustrations**	
		Planning for the end of life (C)	“I want to remain at home as long as possible but if I have serious symptoms I would have to go to hospital.”^57^
			“I think that [hospice] would be a more peaceful place.”^57^
		Dealing with death and dying (U)	“Well, you have to get along with it. At my age [100 years] I expect to die every day.”^65^
		Faith, belief and existential issues (U)	“I’ve had more lives than a cat in the last 26 years. I should have been dead more times than a cat. I’ve had more than 9 lives.”^55^
		Awareness that cancer and death are controlled by a higher power (U)	“The Lord knew I had cancer even before I did. Already knew what I had before the storms [Hurricane Katrina]. And He wanted me to be in a place [Texas] where He knew I could be taken care of. All the storms were putting me in another a place where He knew I could be taken care of. I don’t know when You [God] gonna decide to take me away from here. It may not be the cancer that takes me. That’s up to Your [God’s] hand.”^64^
		Concern about the dying process and death (U)	“I know I’m dying. I just hope it doesn’t take too long.”^70^
		Musings on death (U)	“I used to be afraid of death, now I am not feeling that. One of my superiors has said that death is not just the end of the road; it is just the bend of the road.”^59^
		Individual death pathways (C)	“To be patient.”^99^
		**Quantitative findings** ^a^	
		Fear of death	Bereavement support
		Feelings about death and dying	Death and resolution
		Difficulties concerning the meaning of death	Need for continuity and an afterlife
		Dying and death	To have my funeral arrangements made
		Dealing with spiritual issues of death and dying	
	Need to die in a preferred way	**Qualitative findings and illustrations**	
		To receive assistance in facing death peacefully (U)	“If there were reincarnations, what could I do to prevent me from becoming a pig in the rebirth?”^66^
		The need for a final resting place for the body (U)	“If I don’t die at home in my own bed after I say goodbye to my family members and close friends, my soul might get lost.”^68^
		**Quantitative findings** ^a^	
		To die at home	Being able to choose the place where you want to die
		Not dying alone	

a Quantitative findings reflect survey items. No further illustrations are given since the primary studies do not provide them.

These synthesized findings represent needs that patients express in the palliative phase of their illness concerning the social and spiritual dimensions and will be discussed in the following paragraphs.

### Synthesized finding 1: Being autonomous

**Figure 2.**
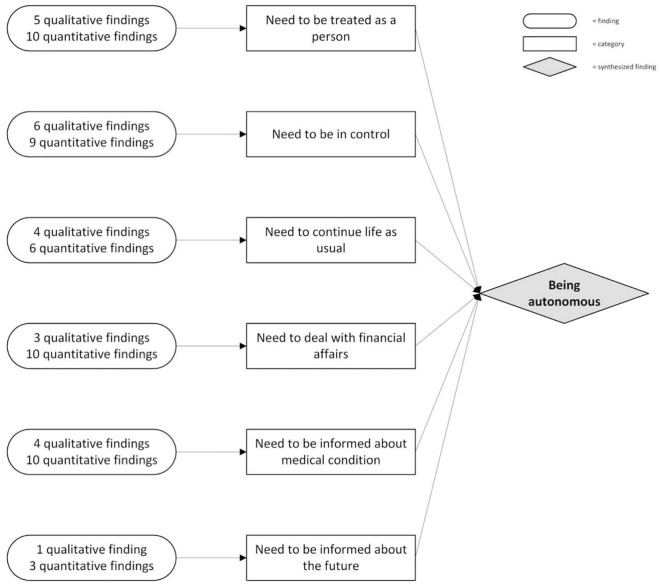
Synthesized finding 1: being autonomous.

Studies showed various ways in which patients express their need to be autonomous—Figure 2 shows which categories of needs constitute this synthesized finding.

Most simply, being autonomous can be explained as being one’s person and one’s capability for self-governance, that is, being directed by desires and wishes that are not imposed externally.^19,100^ Being autonomous touches upon patients’ need to be treated and seen as the person they are,^56,65,68^ and to continue life as usual.^60,67,99^ It also concerns staying in control over their lives,^57,60,64,65,99^ the care they receive,^19^ and their financial concerns.^54,62,73,75,94^ To achieve this, patients expressed the need to be informed about their medical condition^58,61,91,94^ and their future.^58^ Patients’ need for wanting to be autonomous relates to both the social and spiritual dimension: for some, autonomy relates to practicalities, values, and roles; for others, it touches upon their identity and core being, and for some patients, wanting to be autonomous related to all aspects listed above. Needs concerning being autonomous and decision-making are approached differently in Asia and the Western world. Whereas patients in both Asia and the Western world express information needs, these needs are purposefully neglected by family and healthcare professionals of Asian patients.^66,68^ Patients do express they appreciate to open communication about how their disease and situation affects their life.

### Synthesized finding 2: Being connected

**Figure 3.**
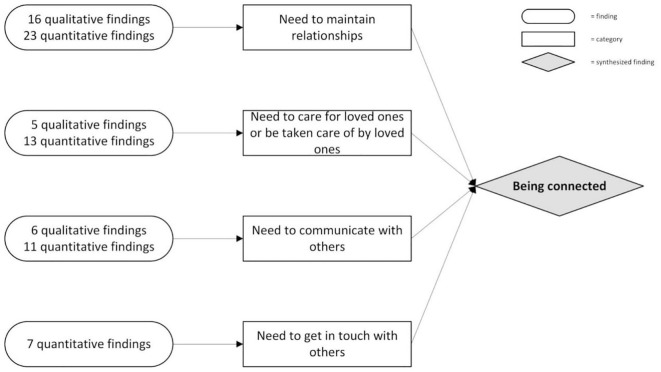
Synthesized finding 2: being connected.

Findings concerning needs for relationships, love, affection, care, communication and support were abundant throughout the literature. Being connected was of overall importance. Figure 3 shows the construction of this synthesized finding.

Being connected appears to be of universal importance throughout all cultures covered by the included literature and is related to both the social and the spiritual dimension. Patients expressed the desire to be connected to family, friends and other loved ones,^56,57,65–69,73,75,89,94,99^ to a divine entity,^63,73–76,85,87^ and healthcare professionals.^65^ Religious connection is a way of being connected and is therefore not seen as a distinct need. Being connected is expressed socially as a need for social support,^57,96^ financial support,^66^ and social relations.^57^ Spiritually, being connected concerned compassion, love and respect,^63^ a sense of (religious) belonging^63–65,69^ and a sense of being more than an individual.^82,84^

### Synthesized finding 3: Finding and having meaning

**Figure 4.**
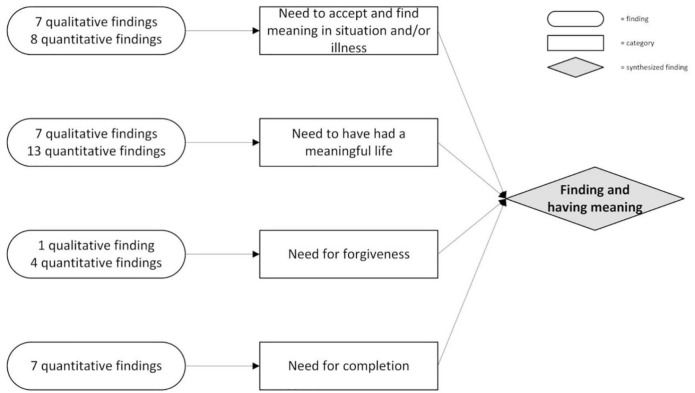
Synthesized finding 3: finding and having meaning.

Patients expressed the need for meaning in the context of their disease.^57,75,84^ The construction of this synthesized finding is depicted in Figure 4.

Patients found and had meaning in the past, present and future. Firstly, patients expressed the need to feel their life has been meaningful,^55,73,74^ to remember their accomplishments,^75,96^ and to feel their lives were complete.^75^ Secondly, patients needed to find meaning in the present: in their disease,^56,81,85,89^ their experience,^89^ in nature and beauty,^82,85^ or through religion and religious rituals.^56,67,69,74,87^ Lastly, patients needed to find meaning in what had yet to come: they needed to complete their life through finishing or resolving unfinished business,^73–75,85,96^ and through setting new priorities in life.^56,94^ Having a meaningful life was lastly expressed through the need for forgiveness: forgiving others^87^ or being forgiven by others and being free from guilt and blame.^66,74,87^ The need for forgiveness and the need to complete life were mainly expressed in quantitative studies. Only in one Asian study was the need for forgiveness found through qualitative inquiry.^56^

### Synthesized finding 4: Having a positive outlook

**Figure 5.**
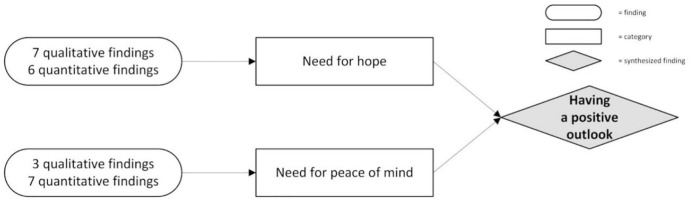
Synthesized finding 4: having a positive outlook.

In the context of being faced with a life-limiting illness, patients expressed the need for a positive outlook through hope and peace of mind. Figure 5 shows how this synthesized finding is constructed.

Patients spoke of hope as a positive outlook or positive thinking,^66,82,89,99^ reassurance,^67^ strength,^65^ peace of mind,^56,85^ and the wish that everything would end well.^84^ They found hope through reassurances by healthcare professionals,^66^ being told the truth about their situation^55,66^ or knowing healthcare professionals have done all they could.^55,73^ Staying strong also demanded inner peace from these patients.^56,61,74,85^

### Synthesized finding 5: Dealing with dying and death

**Figure 6.**
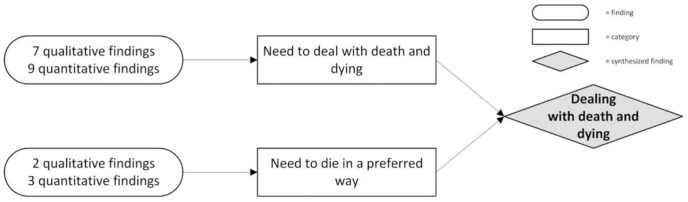
Synthesized finding 5: dealing with death and dying.

Patients indicate the importance of addressing the uncertainties and feelings that come with dealing with dying and death. Figure 6 shows how this synthesized finding is constructed.

Patients expressed that they needed to deal with their spiritual issues and concerns about death and dying^82,94,99^ and difficulties concerning death’s meaning.^76,81^ Also, patients needed to reflect on the possibility of an afterlife.^82,84,85,87^ Patients expressed that they sometimes wanted to have conversations with a healthcare chaplain or other healthcare professionals about their impending death and its meaning.^67,75,85,93^ For some patients, it was vital that they could relate to their death through their religion.^61,66^ Patients also expressed socials needs considering their death: staying at home as long as possible,^57^ planning the funeral^64,75^ and what should happen with their bodies after they died.^64^ Moreover, patients expressed that they wanted to die in their preferred way. For some, this was a spiritual need, wanting to die with peace of mind.^61,66^ For others, this had to do with not dying alone or wanting to die at a preferred location, such as their own home.^73,75^

## Discussion

This study aimed to gain insight into the social and spiritual needs of patients with a life-limiting illness and distinguish between these needs. This systematic review confirmed that these patients report various needs in the palliative phase of their illness, some of which are categorized as either social or spiritual needs. These needs can be summarized in five synthesized findings: being autonomous, being connected, having meaning, having a positive outlook, and dealing with death and dying.

These synthesized findings are consistent with literature focusing on patients’ social and spiritual needs. Primary studies, however, focus either on patients’ palliative care needs in general^54,57,58,60,62,64,65,69–71^ or on specifically patients’ spiritual needs,^55,56,59,63,66–68,72,74,82,84,85,87^ and focus less on social needs^61^ as the primary outcome. Measurement instruments used in survey studies often pay less attention to social needs than they do to spiritual needs.

Looking at the synthesized findings, it becomes apparent that the expressed needs and analytical themes encompass both social and spiritual needs. “Being autonomous”, “being connected,” and “dealing with death and dying” encompass social and spiritual needs, whereas “having meaning” and “having a positive outlook” lean more toward spiritual needs alone.

### Strengths and limitations

This study’s first strength is the extensive search using a search strategy that focused on “spiritual needs,” “social needs,” and related terminology. In contrast to other reviews, the aim was not to identify needs but to synthesize and aggregate the themes other studies identified.^31^ Next, a multidisciplinary team of experienced researchers performed the analysis, enabling them to look at the results from different disciplines’ perspective and present them accordingly. Another strength lies in the inclusion of qualitative and quantitative studies, enabling the research team to cover the entire research field’s scope. The resulting meta-aggregation permitted the researchers to assess the themes that previous studies addressed and identified.^47^ The use of an integrated design allowed both qualitative and quantitative studies in the meta-aggregation.^34^ Previous reviews only incorporated qualitative studies.^30–32 (Cagle, Bunting and Kelemen, 2017).^

The choices that were made came with some setbacks. Firstly, this review’s search strategy included terms such as “social” and “spiritual,” thereby already narrowing the search in advance. Patients themselves do not frame their needs in this way. Therefore, a search strategy only including “needs” would have been more transparent. However, initial searches resulted in over 30,000 results this way, making the study unmanageable. Therefore, the research team decided against this option. Secondly, seeing patients’ needs in light of either the social or spiritual dimension will always be based on interpretations by the researchers conducting the analysis. Since definitions of the social and spiritual dimensions overlap, associating needs with these dimensions can always be disputed. We minimized this bias by interpreting reported needs in a multidisciplinary research team with shared expertise and competencies in chaplaincy and nursing and establishing a partnership with a delegation of social workers. Lastly, although the integrative design suits the research question well in this study, it also comes with limitations. This design is inherently less reproducible and transparent due to the design’s iterative nature. There may also be some issues concerning the combining of qualitative and quantitative methodologies of the included studies. While by qualitizing the data from quantitative studies, the findings become comparable, one might still oppose the design’s assumption that methodological differences can be minimized.^101^

### Socio-spiritual approach

As mentioned above, the synthesized findings reflect patients’ social and spiritual needs. However, patients themselves do not label their needs as “social” or “spiritual.” Needs often comprise multiple dimensions, including the social and spiritual dimension. Consequently, expressed needs are open to interpretation: the same expression might refer to both a social and a spiritual need depending on the patient’s point of view. Therefore, patients’ needs should not be interpreted based on their first expression. An in-depth exploration of these needs is necessary to recognize and understand, and subsequently, distinguish the underlying dimensions to provide appropriate care.

Within the framework of multidimensional symptom management, we propose a socio-spiritual approach to patients’ needs. This approach involves regarding social and spiritual needs as tantamount on a linguistic level but warrants distinguishing them when explored in-depth. This approach honors and preserves the real-life multidimensionality of patients’ needs and raises awareness of the linguistic similarities in the expression of social and spiritual needs.

The socio-spiritual approach fits and elucidates the first step in the method of palliative reasoning: mapping patients’ problems.^102^ Physical and psychological symptoms and needs are often clearly expressed by patients and clearly understood by healthcare professionals. However, social and spiritual needs share a common terminology making it complex to understand them based on their expression alone. Therefore, in-depth exploration of social and spiritual needs is necessary for answering the question “what does it mean?” – as is the case for all multidimensional needs – and for answering an even more fundamental question “what does this mean to you?”.

Adopting a socio-spiritual approach to identifying patients’ needs benefits both patients and healthcare professionals. Patients can express their needs in the way they seem fit and healthcare professionals can assess these needs holistically. This makes sure that the interpretation of patients’ needs is not prematurely reduced to categories that do not fully match with what is at stake for them. Next, being aware of the overlap between social and spiritual needs can encourage healthcare professionals to explore patients’ needs, thus guaranteeing appropriate interventions.

As such, the socio-spiritual approach facilitates and demands interdisciplinary teamwork to combine healthcare professionals’ expertise to decide which discipline is best suited to meet the needs.

### Recommendations for healthcare practice and future research

Based on the findings of this review, the socio-spiritual approach is recommended in multidimensional symptom management. The overlap in the expression of social and spiritual needs can create confusion in day-to-day care. Therefore, social and spiritual needs should be carefully assessed and interpreted by the healthcare team to assign patient-tailored care. To understand and distinguish between patients’ social and spiritual needs and facilitate patient-tailored care, healthcare professionals of all disciplines require evidence-based information on exploring, assessing, and recognizing the overlap between these needs.^74,82,103^

Patient Reported Outcome Measures (PROMs) can help patients think about their own needs and wishes and help focus on what matters at the moment, supporting the in-depth exploration.^74^ The use of PROMs can reinforce patient autonomy and improve patient-professional communication and communication between professionals. Furthermore, PROMs support monitoring needs throughout time, especially when continuity in day-to-day care cannot be guaranteed in person due to part-time work and irregular work hours.^104,105^

Hence, more clinical applicable PROMs should be developed and employed, for example, PROMs that are not too demanding for patients or too extensive in their setup, as is the case for research purposes. The Utrecht Symptom Diary – 4 Dimensional (USD-4D) is a PROM used in palliative care to signal and monitor patients’ symptoms and needs in all four dimensions: physical, psychological, social, and spiritual. Five items concern both the social and spiritual dimensions. A recent study showed that patients interpret these items as either social and spiritual, or both, depending on their context.^106^ Since patients use the same vocabulary to express their social and spiritual needs, healthcare professionals should always be aware that they explore what patients mean when expressing their needs. As a result, patients can indicate how they interpret these items. This, in turn, helps healthcare professionals in appointing the appropriate disciplines when an intervention is necessary.

Future research should focus on patients’ needs being multidimensional and not referring to solely one dimension. This review showed how this holds for social and spiritual needs. In the long run, this could lead to the use of PROMs in which the physical, psychological, social, and spiritual dimensions are integrated so that patients’ needs are not drawn apart in advance along the boundaries of dimensions. Furthermore, more research should focus on the social dimension of palliative care as a separate domain.

## Conclusion

This review identified five synthesized findings that encompass patients’ social and spiritual needs: being autonomous, being connected, having meaning, having a positive outlook, and dealing with death and dying. These findings were synthesized from both qualitative and quantitative studies.

Patients do not distinguish between social and spiritual needs themselves. However, healthcare professionals should do so to allocate appropriate, patient-tailored care. The socio-spiritual approach to patients’ needs raises awareness about linguistic similarities in expression between social and spiritual needs that should be explored in-depth. Hence, this approach honors and preserves the multidimensionality of patients’ needs and enables comprehensive palliative care. Clinical applicable multidimensional PROMs can support identifying and exploring patients’ social and spiritual needs.

## Supplemental Material

sj-docx-1-pmj-10.1177_02692163211010384 – Supplemental material for Toward a socio-spiritual approach? A mixed-methods systematic review on the social and spiritual needs of patients in the palliative phase of their illness Click here for additional data file. Supplemental material, sj-docx-1-pmj-10.1177_02692163211010384 for Toward a socio-spiritual approach? A mixed-methods systematic review on the social and spiritual needs of patients in the palliative phase of their illness by Tom Lormans, Everlien de Graaf, Joep van de Geer, Frederieke van der Baan, Carlo Leget and Saskia Teunissen in Palliative Medicine
