# Supplementary material for: Toward a socio-spiritual approach? A mixed-methods systematic review on the social and spiritual needs of patients in the palliative phase of their illness
Source: Palliat Med. 2021 Apr 20;35(6):1071–98. doi: 10.1177/02692163211010384 (PMC8189005; doi:10.1177/02692163211010384)
Supplement: sj-docx-1-pmj-10.1177_02692163211010384 – Supplemental material for Toward a socio-spiritual approach? A mixed-methods systematic review on the social and spiritual needs of patients in the palliative phase of their illness [file sj-docx-1-pmj-10.1177_02692163211010384.docx]

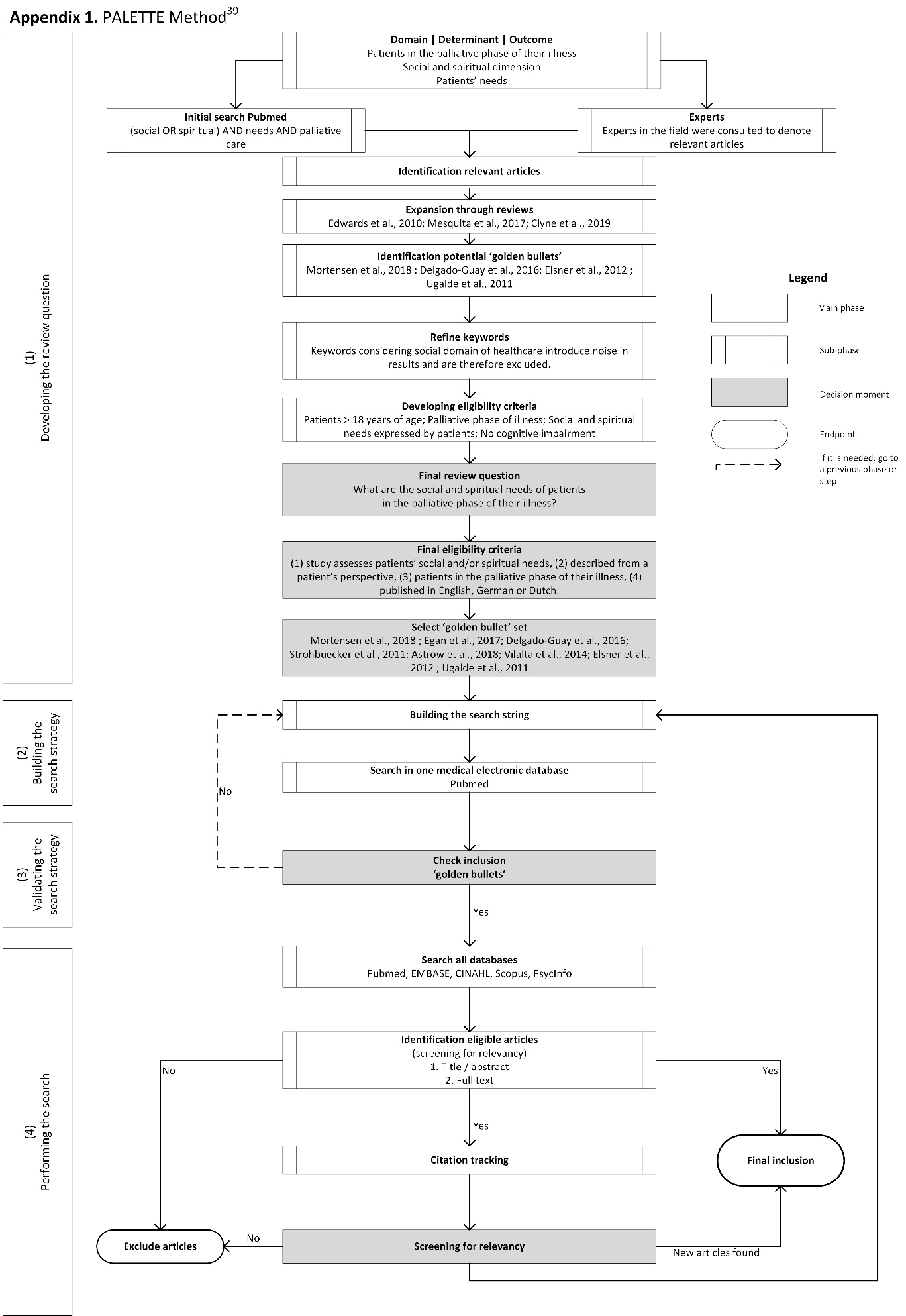
**Appendix 2.** Adapted searchstrings

Searchstring Embase

| Palliative care | #1 | **‘Palliative therapy’/exp OR ’terminal care’/exp** |
| --- | --- | --- |
|  | #2 | ‘palliative care’:ab,ti OR ‘hospice care’:ab,ti OR ‘supportive care’:ab,ti OR ‘terminal care‘:ab,ti OR ‘end of life’:ab,ti OR ‘advanced cancer’:ab,ti OR ‘terminal cancer’:ab,ti |
|  | #3 | #1 OR #2 |
| Dimension | #4 | **’Religion ’/exp OR ’social care’/exp OR ‘social psychology’/exp** |
|  | #5 | ‘religion‘:ab,ti OR ‘religiosity‘:ab,ti OR ‘religious‘:ab,ti OR ‘faith‘:ab,ti OR ‘spiritual‘:ab,ti OR ‘spirituality‘:ab,ti OR ‘existential‘:ab,ti OR ‘social‘:ab,ti OR ‘psychosocial‘:ab,ti OR ‘spirit’:ab,ti OR ‘soul’:ab,ti OR ‘meditation’:ab,ti OR ‘pray’:ab,ti OR ‘rite’:ab,ti OR ‘divine’:ab,ti or ‘god’:ab,ti OR ‘church’:ab,ti OR ‘dignity’:ab,ti OR ‘hope’:ab,ti OR ‘wellbeing’:ab,ti OR ‘social support’:ab,ti OR ‘family’:ab,ti OR ‘network’:ab,ti ‘family network’:ab,ti OR ‘connection’:ab,ti OR ‘spouse’:ab,ti OR ‘empathy’:ab,ti |
|  | #6 | #4 OR #5 |
| Needs | #7 | **‘Health care need’/exp OR ’needs assessment’/exp** |
|  | #8 | 'need':ab,ti OR 'issue':ab,ti OR 'experience':ab,ti OR 'dilemma':ab,ti OR 'wish':ab,ti OR 'demand':ab,ti OR 'burden':ab,ti OR 'preference':ab,ti OR ‘unmet’:ab,ti |
|  | #9 | #7 OR #8 |
| Population | #10 | **'Patient'/exp OR 'attitude'/exp OR 'patient satisfaction'/exp OR 'patient reported outcome'/exp** |
|  | #11 | ‘patient‘:ab,ti OR ‘patients‘:ab,ti |
|  | #12 | #10 OR #11 |
|  |  |  |
| Final search | #13 | #3 AND #6 AND #9 AND #12 |

Searchstring Psychinfo

| Palliative care | #1 | **(Palliative therapy OR terminal care).mh** |
| --- | --- | --- |
|  | #2 | Palliative care.ab. OR hospice care.ab. OR supportive care.ab. OR terminal care.ab. OR end of life.ab. OR advanced cancer.ab. OR terminal cancer.ab. OR palliative care.ti. OR hospice care.ti. OR supportive care.ti. OR terminal care.ti. OR end of life.ti. OR advanced cancer.ti. OR terminal cancer.ti. |
|  | #3 | #1 OR #2 |
| Dimension | #4 | **(Religion OR social behavior OR psychology).mh** |
|  | #5 | Religion.ab. OR religiosity.ab. OR religious.ab. OR faith.ab. OR spiritual.ab. OR spirituality.ab. OR existential.ab. OR social.ab. OR psychosocial.ab. OR spirit.ab. OR soul.ab. OR meditation.ab. OR pray.ab. OR rite.ab. OR divine.ab. or god.ab. OR church.ab. OR dignity.ab. OR hope.ab. OR well being.ab. OR social support.ab OR family.ab. OR network.ab. OR family network.ab. OR connection.ab. OR spouse.ab. OR empathy.ab. OR religion.ti. OR religiosity.ti. OR religious.ti. OR faith.ti. OR spiritual.ti. OR spirituality.ti. OR existential.ti. OR social.ti. OR psychosocial.ti. OR spirit.ti. OR soul.ti. OR meditation.ti. OR pray.ti. OR rite.ti. OR divine.ti. or god.ti. OR church.ti. OR dignity.ti. OR hope.ti. OR well being.ti. OR social support.ti OR family.ti. OR network.ti. OR family network.ti. OR connection.ti. OR spouse.ti. OR empathy.ti. |
|  | #6 | #4 OR #5 |
| Needs | #7 | **Needs assessment.mh** |
|  | #8 | Need.ab. OR issue.ab. OR experience.ab. OR dilemma.ab. OR wish.ab. OR demand.ab. OR burden.ab. OR preference.ab. OR unmet.ab. OR need.ti. OR issue.ti. OR experience.ti. OR dilemma.ti. OR wish.ti. OR demand.ti. OR burden.ti. OR preference.ti. OR unmet.ti. |
|  | #9 | #7 OR #8 |
| Population | #10 | **(Patients OR attitude to death OR patient satisfaction OR Patient reported outcome measures).mh** |
|  | #11 | Patient.ab. OR patients.ab. OR patient.ti. OR patients.ti. |
|  | #12 | #10 OR #11 |
|  |  |  |
| Final search | #13 | #3 OR #6 OR #9 OR #12 |

Searchstring Cinahl

| Palliative care | #1 | **MH Palliative therapy OR MH terminal care** |
| --- | --- | --- |
|  | #2 | TI palliative care OR TI hospice care OR TI supportive care OR TI terminal care OR TI end of life OR TI advanced cancer OR TI terminal cancer OR AB palliative care OR AB hospice care OR AB supportive care OR AB terminal care OR AB end of life OR AB advanced cancer OR AB terminal cancer |
|  | #3 | #1 OR #2 |
| Dimension | #4 | **MH Religion OR MH social behavior OR MH psychology, social** |
|  | #5 | TI religion OR TI religiosity OR TI religious OR TI faith OR TI spiritual OR TI spirituality OR TI existential OR TI social OR TI psychosocial OR TI psychological OR TI psychology OR TI spirit OR TI soul OR TI meditation OR TI pray OR TI rite OR TI divine or TI god OR TI church OR TI dignity OR TI hope OR TI well being OR TI social support OR TI family OR TI network OR TI family network OR TI connection OR TI spouse OR TI empathy OR AB religion OR AB religiosity OR AB religious OR AB faith OR AB spiritual OR AB spirituality OR AB existential OR AB social OR AB psychosocial OR AB psychological OR AB psychology OR AB spirit OR AB soul OR AB meditation OR AB pray OR AB rite OR AB divine or AB god OR AB church OR AB dignity OR AB hope OR AB well being OR AB social support OR AB family OR AB network OR AB family network OR AB connection OR AB spouse OR AB empathy |
|  | #6 | #4 OR #5 |
| Needs | #7 | **MH needs assessment** |
|  | #8 | TI need OR TI issue OR TI experience OR TI dilemma OR TI wish OR TI demand OR TI burden OR TI preference OR TI unmet OR AB need OR AB issue OR AB experience OR AB dilemma OR AB wish OR AB demand OR AB burden OR AB preference OR AB unmet |
|  | #9 | #7 OR #8 |
| Population | #10 | **MH Patients OR MH attitude to death OR MH patient satisfaction OR MH Patient reported outcome measures** |
|  | #11 | TI Patient OR TI patients OR AB patient OR AB patients |
|  | #12 | #10 OR #11 |
|  |  |  |
| Final search | #13 | #3 OR #6 OR #9 OR #12 |

Search string Scopus

| Palliative care | #1 | **KEY ( "palliative care" OR "terminal care" )** |
| --- | --- | --- |
|  | #2 | ( TITLE ( “palliative care” OR “hospice care” OR “supportive care” OR “terminal care” OR “end of life” OR “advanced cancer” OR “terminal cancer”) OR ABS ( “palliative care” OR “hospice care” OR “supportive care” OR “terminal care” OR “end of life” OR “advanced cancer” OR “terminal cancer”) ) |
|  | #3 | #1 OR #2 |
| Dimension | #4 | **KEY ( "religion" OR "medicine and religion" OR "psychology and religion" OR "quality of life" OR "chaplaincy service, hospital" )** |
|  | #5 | ( TITLE ( “religion” OR “religiosity” OR “religious” OR faith” OR “spiritual” OR “spirituality” OR “existential” OR “social” OR “psychosocial” OR “psychological” OR “psychology” OR “spirit” OR “soul” OR “meditation” OR “pray” OR “rite” OR “divine” or “god” OR “church” OR “dignity” OR “hope” OR “well being” OR “social support” OR “family” OR “network” OR “family network” OR “connection” OR “spouse” OR “empathy”) OR ABS ( “religion” OR “religiosity” OR “religious” OR faith” OR “spiritual” OR “spirituality” OR “existential” OR “social” OR “psychosocial” OR “psychological” OR “psychology” OR “spirit” OR “soul” OR “meditation” OR “pray” OR “rite” OR “divine” or “god” OR “church” OR “dignity” OR “hope” OR “well being” OR “social support” OR “family” OR “network” OR “family network” OR “connection” OR “spouse” OR “empathy”) ) |
|  | #6 | #4 OR #5 |
| Needs | #7 | **KEY (“needs assessment”)** |
|  | #8 | ( TITLE ( “need” OR “issue” OR “experience” OR “dilemma” OR “wish” OR “demand” OR “burden” OR “preference” OR “unmet” ) OR ABS ( “need” OR “issue” OR “experience” OR “dilemma” OR “wish” OR “demand” OR “burden” OR “preference” OR “unmet”) ) |
|  | #9 | #7 OR #8 |
| Population | #10 | **KEY (“attitude to death” OR “attitude to health” OR “assessments, patient outcomes”)** |
|  | #11 | ( TITLE ( "patient" OR "patients" ) OR ABS ( "patient" OR "patients" ) ) |
|  | #12 | #10 OR #11 |
|  |  |  |
| Final search | #13 | #3 OR #6 OR #9 OR #12 |
